# Altered microRNA expression profile during epithelial wound repair in bronchial epithelial cells

**DOI:** 10.1186/1471-2466-13-63

**Published:** 2013-11-05

**Authors:** Aleksandra Szczepankiewicz, Peter M Lackie, John W Holloway

**Affiliations:** 1 Laboratory of Molecular and Cell Biology, Department of Pediatric Pulmonology, Allergy and Clinical Immunology, Poznan University of Medical Sciences, 27/33 Szpitalna St., 60-572 Poznan, Poland; 2 Clinical and Experimental Sciences, Faculty of Medicine, University of Southampton, Southampton, UK; 3 Human Development and Health, Faculty of Medicine, University of Southampton, Southampton, UK

**Keywords:** Epithelial cells, Wound repair, miRNA, Profiling, Cluster analysis, Pathway analysis

## Abstract

**Background:**

Airway epithelial cells provide a protective barrier against environmental particles including potential pathogens. Epithelial repair in response to tissue damage is abnormal in asthmatic airway epithelium in comparison to the repair of normal epithelium after damage. The complex mechanisms coordinating the regulation of the processes involved in wound repair requires the phased expression of networks of genes. Small non-coding RNA molecules termed microRNAs (miRNAs) play a critical role in such coordinated regulation of gene expression. We aimed to establish if the phased expression of specific miRNAs is correlated with the repair of mechanically induced damage to the epithelium.

**Methods:**

To investigate the possible involvement of miRNA in epithelial repair, we analyzed miRNA expression profiles during epithelial repair in a cell culture model using TaqMan-based quantitative real-time PCR in a TaqMan Low Density Array format. The expression of 754 miRNA genes at seven time points in a 48-hour period during the wound repair process was profiled using the bronchial epithelial cell line 16HBE14o^-^ growing in monolayer.

**Results:**

The expression levels of numerous miRNAs were found to be altered during the wound repair process. These miRNA genes were clustered into 3 different patterns of expression that correlate with the further regulation of several biological pathways involved in wound repair. Moreover, it was observed that expression of some miRNA genes were significantly altered only at one time point, indicating their involvement in a specific stage of the epithelial wound repair.

**Conclusions:**

In summary, miRNA expression is modulated during the normal repair processes in airway epithelium *in vitro* suggesting a potential role in regulation of wound repair.

## Background

The airway epithelium has been recognized to play a central role in the integration of innate and adaptive immune responses [1-4]. The airway epithelium is also crucial to the origin and progression of respiratory disorders such as asthma, chronic obstructive pulmonary disease, cystic fibrosis and pulmonary fibrosis. In asthma, chronic airway inflammation underlies aberrant repair of the airway that subsequently leads to structural and functional changes in the airway wall. This remodeling is responsible for a number of the clinical characteristics of asthmatic patients.

Normal epithelial repair occurs in a series of overlapping stages. Damage to the epithelium or challenge associated with damage can result in loss of structural integrity or barrier function and local mucosal activation [5]. Studies in animals have shown that the repair of normal airway epithelium after minor damage involves the migration of the remaining epithelial cells to cover the damaged area. This is a rapid process, suggesting an autonomous response by cells in the vicinity of the damage [6]. It includes an acute inflammatory response, with recruitment of immune cells as well as epithelial spreading and migration stimulated by secreted provisional matrix. Once the barrier is reformed, the differentiated characteristics are then restored. The regulation of these processes require complex sequential changes in the epithelial cell biology driven by the phased expression of networks of genes [7].

One biological mechanism that plays a critical role in the coordinate regulation of gene expression such as that required during epithelial wound repair is the expression of small non-coding RNA molecules termed microRNAs (miRNAs) [8]. To date, more than 1000 human miRNAs have been identified [http://microrna.sanger.ac.uk], with documented tissue-specific expression of some of these miRNAs in lung and involvement in the development of lung diseases including lung cancer, asthma and fibrosis [9-15]. MiRNAs have been demonstrated to play a crucial role in epithelial cell proliferation and differentiation [16-18]. The expression in lung epithelium of Dicer, the enzyme responsible for processing of miRNA precursors, is essential for lung morphogenesis [16] and there is differential expression of miRNAs during lung development [17]. Furthermore, transgenic over-expression of miR-17-92 (shown to be over-expressed in lung cancer) in the lung epithelium promotes proliferation and inhibits differentiation of lung epithelial progenitor cells [18]. Recently, it has been reported that miRNA-146a modulates survival of bronchial epithelial cells in response to cytokine-induced apoptosis [19]. In experimental studies, mice lacking miR-155 demonstrated autoimmune phenotypes in the lungs with increased airway remodeling and leukocyte invasion, phenotypes similar to those observed in asthma [20,21].

While a number of studies have examined the role of miRNA in lung development and in disease [9-15], their influence on the regulation of gene expression involved in epithelial wound repair remains unresolved and comprehensive studies on miRNA involvement in epithelial repair and the pathogenesis of airway remodeling are lacking. However in the skin, miRNAs were found to play a crucial role in wound closure by controlling migration and proliferation of keratinocytes in an *in vitro* model of wound repair [22].

Thus the hypothesis of the study was that the stages of wound repair in respiratory epithelium are regulated by the phased expression of specific miRNA species. The aim was to investigate the possible involvement of miRNAs by examining their expression profile in epithelial repair in a cell culture model. Understanding the effect of altered miRNA activity on protein expression during repair processes can be further used to identify pathways targeted by miRNAs that regulate epithelial wound repair, potentially providing a novel therapeutic strategy for asthma and other respiratory diseases with underlying aberrant epithelial wound repair.

## Methods

### Cell culture and wounding assays

The 16HBE14o- bronchial epithelial cell line was cultured under standard conditions [23]. For the wounding assay, cells were seeded on 6-well plates at the initial density of 3x10^5^ cells and cultured until confluent. Forty eight hours after reaching full confluence cells were damaged by scraping off the monolayer with a hatch-cross wounding pattern using a P200 Gilson pipette tip. After that, the medium and cell debris were removed by pipetting off the medium and 2 ml of fresh serum-containing medium was added to the remaining cells. For all experiments, at least two points of reference per well of a 6-well plate were used for post-injury analyses. Several time-lapse experiments were performed to establish consistent experimental conditions and the timing of the stages of wound repair.

### Time lapse microscopy

Time lapse images were captured at 15 minute intervals on a Leica DM IRB phase-contrast inverted microscope (Leica; Milton Keynes, UK) in a chamber maintained at 36 ± 1°C and 5% CO_2_ atmosphere. The images were collected with a cooled Hamamatsu ORCA digital camera (Hamamatsu Photonics, Welwyn Garden City, UK) connected to a computer running Cell^P software (Olympus, London, UK) for 30 hours (ensuring complete wound closure is included in the time course). For quantitative analysis of the area of damage and hence ongoing repair in time lapse serial images ImageJ software [24] was used.

### RNA isolation

RNA isolation was performed with the use of an Exiqon RNA isolation kit. Samples were collected in triplicate for each of the following time points: baseline, 2, 4, 8, 16, 24 and 48 hours after wounding. RNA isolation was performed according to the manufacturer’s protocol from 6-well plates (9.5 cm^2^ of growth area) and the amount of starting material was 1×10^6^ cells per well. Samples were frozen at -70°C for subsequent use in microarray experiments.

### RNA quality control

The concentration of total RNA in each sample was determined using a NanoDrop 1000 spectrophotometer. The integrity of total RNA extracted was assessed by a Lab901 Gene Tools System. The passing criteria for use in microarrays was: sample volume of 10–30 μl, RNA concentration > 50 ng/μl, SDV ≤ 3.1 (ScreenTape Degradation Value), which corresponds to RIN ≥ 9.0, purity: OD 260/280 > 1.7, OD 260/230 > 1.4.

### Micro RNA profiling

Micro-RNA expression profiling of bronchial epithelial cells was performed in three replicates per time point following wounding. TaqMan Array Human MicroRNA Card A and B (Applied Biosystems) (based on Sanger miRBase 9.2) was utilised for specific amplification and detection of 754 mature human miRNAs by TaqMan-based quantitative real-time PCR in a TaqMan Low Density Array format (TLDA) using TaqMan MicroRNA Reverse Transcription Kit and Megaplex RT Primers (Human Megaplex™ RT Primers Pool A and B). The resulting cDNA combined with TaqMan Universal PCR Master Mix, No AmpErase UNG was loaded into the arrays and TaqMan real-time PCR was performed using the 7900HT Fast Real-Time PCR System (Applied Biosystems) according to the manufacturer’s protocol.

Raw data were obtained using SDS 2.3 software (Applied Biosystems). All SDS files were analyzed utilizing the RQ Manager 1.2 software (Applied Biosystems). The comparative analysis of miRNA expression datasets between baseline and each time point following the wounding assay was performed using DataAssist software v.3.01 (Applied Biosystems). The comparative CT method [25] was used for calculating relative quantitation of gene expression after removing outliers with use of Grubbs’ outlier test together with a heuristic rule to remove the outlier among technical replicates and data normalization was based on the endogenous control gene expression method (U6 snRNA-001973) where the mean of the selected endogenous control was used to normalize the Ct value of each sample.

The data from miRNA profiling have been deposited in ArrayExpress database (accession no. E-MEXP-3986).

### Cluster analysis

To identify the clusters of miRNAs following the same expression profile over time, we performed cluster analysis using STEM (Short Time series Expression Miner) software available at: http://www.cs.cmu.edu/~jernst/stem[26].

### Target genes and pathways prediction

To identify potential common biological pathways for miRNAs showing similar expression profiles in cluster analysis, we performed pathway enrichment analysis. The best predicted candidate mRNA genes for each differentially expressed miRNA were identified using the miRNA BodyMap tool available at: http://www.mirnabodymap.org. The tool enables the selection of target genes based on the use of several prediction algorithms at a time: DIANA, PITA, TargetScan, RNA22 (3UTR), RNA22 (5UTR), TargetScan_cons, MicroCosm, miRDB, RNA22 (5UTR), TarBase and miRecords. To minimize the target prediction noise, only target genes predicted by five or more of the prediction algorithms mentioned above were included.

The lists with predicted target genes were then analysed with use of The Database for Annotation, Visualization and Integrated Discovery (DAVID) v.6.7 [27,28] to identify BioCarta & KEGG pathways [29,30] enriched functional-related gene groups and biological themes, particularly gene ontology (GO) terms [31] in which the analysed sets of target genes were statistically the most overrepresented (enriched).

### Statistics

The statistics applied by Data Assist software for each sample included calculation of the relative quantification (RQ) = 2 (-ΔCt)/2(-ΔCt reference). The standard deviation (SD) was calculated for CT values of each of the three technical replicates and was used to calculate the RQ Min and RQ Max [RQ Min = 2(-ΔCt – SD)/2(-ΔCt reference), RQ Max = 2 (-ΔCt + SD)/2(-ΔCt reference)]. Pearson's product moment correlation coefficient (r) was calculated for CT or ΔCT values of sample pairs as below and plotted on the Signal Correlation Plot and Scatter Plot respectively.

$$r=\frac{N\;\mathrm{\Sigma XY}-\left(\mathrm{\Sigma X}\right)\left(\mathrm{\Sigma Y}\right)}{\sqrt{\mathrm{N\Sigma}X^{2}-{\left(\mathrm{\Sigma X}\right)}^{2\;}}\sqrt{\mathrm{N\Sigma}Y^{2}-{\left(\mathrm{\Sigma Y}\right)}^{2}}}$$

Distances between samples and assays were calculated for hierarchical clustering based on the ΔCT values using Pearson’s correlation or the Eucidian distance calculated as follows [https://products.appliedbiosystems.com]. For a sample pair, the Pearson's product moment correlation coefficient (r) was calculated considering all ΔCT values from all assays, and the distance defined as 1 – r. For an assay pair, r was calculated considering all ΔCT values from all samples and the distance defined as 1 – r. Euclidean Distance calculated as $\sqrt{\Sigma {\left(\;\mathrm{\Delta CT}\;\left(i\right)-\mathrm{\Delta CT}\left(j\right)\right)}^{2}}$ where, for a sample pair, the calculation is done across all assays for sample i and sample j while for an assay pair, the calculation is done across all samples for assay i and assay j.

## Results

### Characterisation of epithelial wound repair model

**Figure 1:**
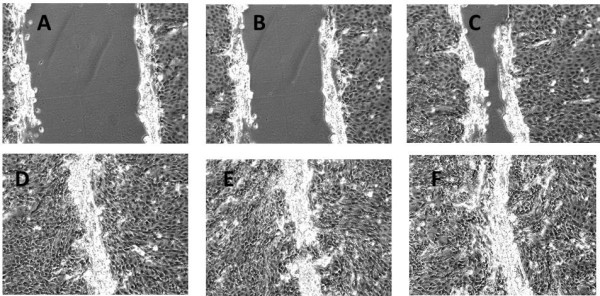
Stages of wound repair at different time points (A – 2 hrs, B – 4 hrs, C – 8 hrs, D – 16 hrs, E – 24 hrs, F – 48 hrs post wounding), n=3 wells for each time point.

To analyse the changes in miRNA expression profile during epithelial wound repair, we used a previously well established *in vitro* model mimicking this process [23,32-34], that allowed real-time monitoring of the rate of epithelial repair and quantitative analysis using time-lapse microscopy. The following time points were selected for miRNA expression profile analysis: baseline immediately before wounding. (A) 2 hours after wounding: cells adjacent to the wound initiate a response but have not migrated substantially. (B) 4 hours after wounding: 25% of the original wound area has been covered by cells. (C) 8 hours after wounding: 50% of wounded area covered by cells. (D) 16 hours after wounding: wounded area completely covered by cells. Once the wound is covered cell proliferation and re-differentiation may still be in progress so additional time points were added. (E) 24 hours post-wounding (F) 48 hours after wounding (Figure 1). With the exception of cells damaged during the original mechanical wounding, cell death was not seen in the repairing areas by time lapse microscopy.

### Global miRNA expression profile altered during epithelial wound repair

Expression profiling analysis revealed a large number of mature miRNAs that were modulated at different time points during epithelial repair with a fold change above 2.0 (Table 1). Numerous miRNAs showed significantly increased or decreased expression (>10-fold) at different time points as compared to baseline (presented in Additional file 1). Based on the high fold change values at different time points, ten miRNAs were found to undergo a significant modulation (both up- or down-regulation) at five or more of the seven time points analysed (Additional file 2). We also observed that the alterations in expression of some miRNA genes were limited to a single time point of wound repair, whereas at the other time points the expression levels did not differ much from the baseline, suggesting their involvement at a particular stage of repair (marked in red in Additional file 1).

**Table 1:** Number of miRNAs with >2.0-fold change in expression at different time points after wounding

	**Time point (post wounding)**					
Mode of miRNA alteration	2 hrs	4 hrs	8 hrs	16 hrs	24 hrs	48 hrs
Upregulated	70	128	85	37	35	252
Downregulated	57	54	80	165	136	23

### Cluster analysis

**Figure 2:**
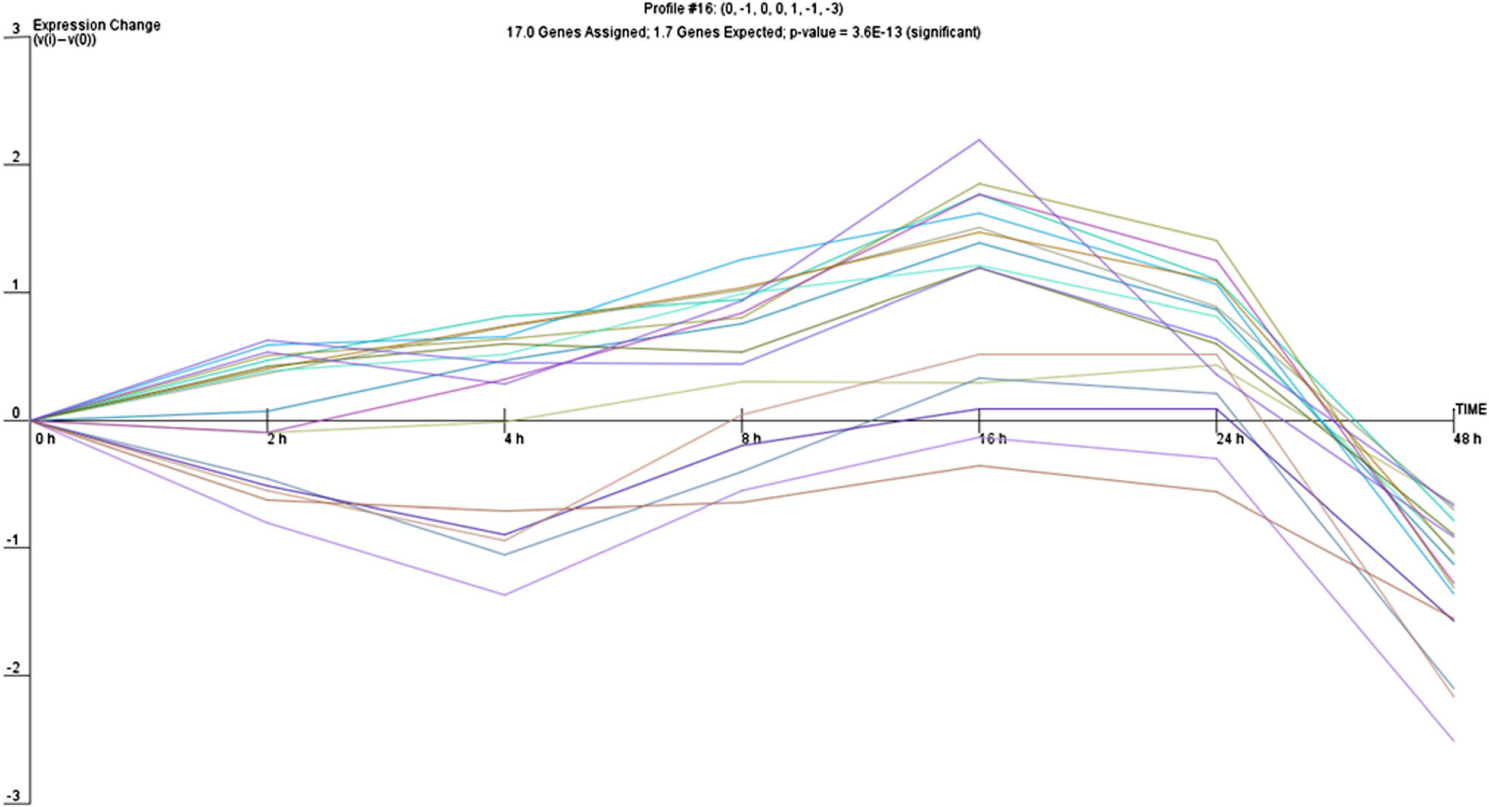
Profile 16 of miRNA with similar expression pattern during wound repair.

**Figure 3:**
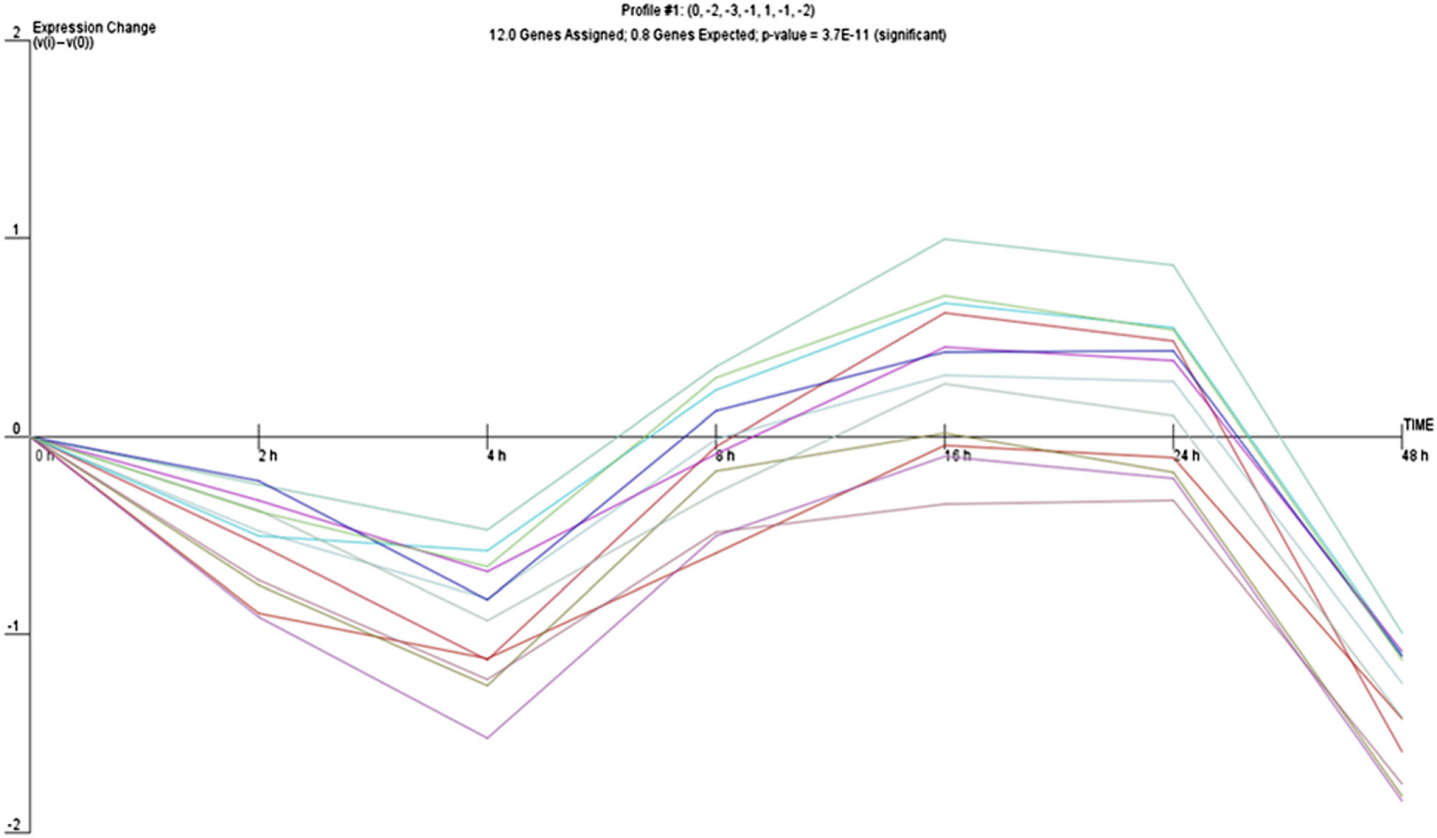
Profile 1 of miRNA with similar expression pattern during wound repair.

**Figure 4:**
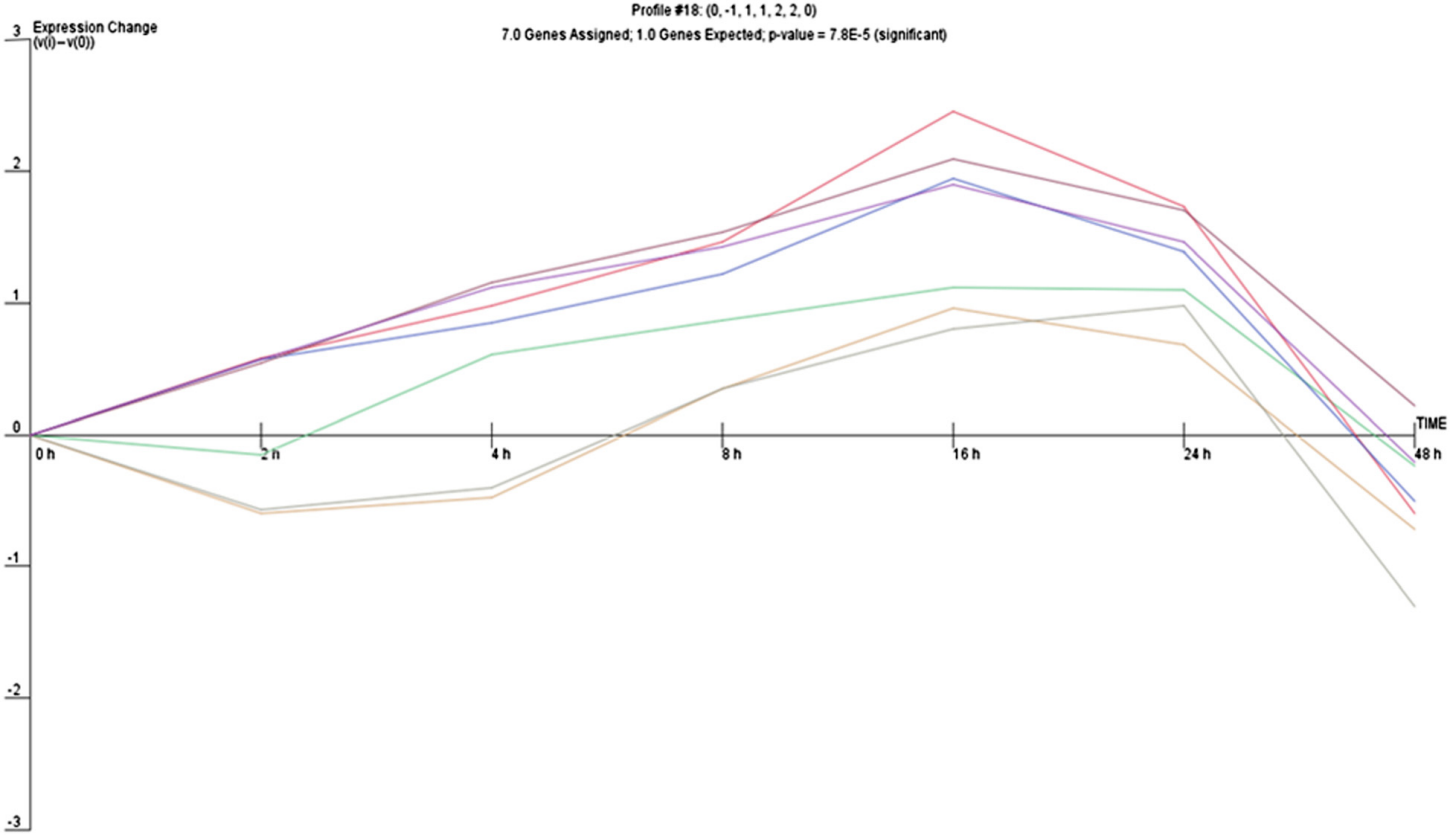
Profile 18 of miRNA with similar expression pattern during wound repair.

We then hypothesized that, given the number of miRNA genes undergoing significant changes during the epithelial repair process, a common expression profile might be shared by miRNAs whose expression is regulated by particular transcriptional activation pathways. Therefore, we analysed the expression of miRNA genes with use of the clustering algorithm STEM [26], assigning each gene passing the filtering criteria to the model profile that most closely matches the gene's expression profile as determined by the correlation coefficient. Since the model profiles are selected by the software by random allocation, independent of the data, the algorithm then determines which profiles have a statistically significant higher number of genes assigned using a permutation test. It then uses standard hypothesis testing to determine which model profiles have significantly more genes assigned as compared to the average number of genes assigned to the model profile in the permutation runs. Our cluster analysis revealed three significant miRNA expression profiles (16, 1 and 18) over 48 hours of wound repair (Figures 2, 3 and 4).

Profile 16 included genes that gradually increase between 2 and 16 hours and then display a sudden drop in expression 16 hours post-wounding, which corresponded to the completion of cell proliferation and the restoration of the monolayer after wounding in time lapse observations. Profile 1 was characterized by significant decrease of miRNA expression 4 hours after wounding followed by a significant increase with a maximum 16 hours post-wounding, suggesting induction of transcription of genes involved in the early response to stress due to mechanical cell damage which are subsequently switched off. Profile 18 shared some similarities with profile 1, although it showed a more gradual decrease in miRNA expression 4 hours post-wounding, that then increased steadily to reach a maximum at 16 hours and afterwards gradually decreased. The different profiles of miRNA expression are shown in Figures 2, 3 and 4. The miRNA genes sharing the common expression pattern during epithelial wound repair are listed in Additional file 3.

### Identification of biological processes regulated by miRNAs

#### Pathways in clusters of miRNAs

The next question to be addressed was if miRNA clusters of characteristic expression profile during epithelial wound repair identified using STEM were regulating target genes from the same biological pathways or processes. To analyse this, we used highly predicted miRNA targets (mRNAs confirmed to be a target of specific miRNA by at least five different algorithms) to create a list of potential miRNA target genes, which were then analysed utilising the DAVID online database for annotation and visualization [27,28]. The use of DAVID enabled the integration of the miRNA target genes into common pathways or GO processes. Analysis of targets predicted for each miRNA expression cluster generated by STEM enabled us to predict four significantly enriched pathways for profile 16, including the neurotrophin signalling pathway, ERBB signalling pathway, MAPK signalling pathway and the RIG-I-like receptor signalling pathway. Six pathways were predicted for the targets of miRNAs demonstrating expression in profile 1: adherence junction, acute myeloid leukaemia, small lung cancer, cell cycle, pathways in cancer and the chemokine signalling pathway. No common pathways were predicted for profile 18. The predicted pathways are shown in Additional files 4 and 5.

For all the profiles, DAVID also predicted numerous biological processes where miRNAs targets play a significant role (see Additional file 6). In general, predicted biological processes and pathways were mainly associated with cell cycle regulation and induction of mitotic divisions, switching on anti-apoptotic genes (ECM, PKB/Akt and IKK) and genes stimulating proliferation (such as MEK, PPARγ) that are of known importance in epithelial wound repair. Apart from well documented biological processes, we also observed that, surprisingly, the most significantly overrepresented were target genes involved in the neurotrophin signalling pathway which suggests its importance in epithelial wound repair process (Additional file 7).

### Target pathways at different stages of wound repair

To identify the most important pathways involved at different stages of epithelial wound repair *in vitro* we also performed pathway enrichment of miRNAs significantly altered only at one time point of wound repair (see Additional file 1, genes in red). For those genes, targets were predicted as above and DAVID was used to identify potential pathways and biological processes. The main observation for epithelial cells in the early phase of repair (2 hours post-wounding) were miRNAs being up-regulated, suggesting switching off target genes and processes associated with response to cellular stress (MAP kinase pathway), regulation of actin cytoskeleton, cell proliferation and migration. The main pathways targeted by up-regulated miRNAs identified for the repair 4 hours after cell damage included genes involved in negative regulation of transcription, RNA metabolism, regulation of cell motion and the cytoskeleton. The most important processes at 8 hours after wounding involved a number of up-regulated miRNAs at this time point and indicating the switching off of genes involved in negative regulation of gene expression and negative regulation of cell communication. At 16 hours following epithelial cell wounding we observed a number of miRNA genes that were down regulated and, therefore, switching on genes involved in mitotic cell cycle, negative regulation of cell death, cell proliferation, ERBB signalling pathway (cell proliferation, survival, migration). This may suggest the predominance of a proliferating phenotype of cells after the damaged area was closed by spreading and migrating cells. After 24 hours post-wounding we observed further down regulation of miRNA genes. Two were of particular interest as they are responsible for switching on genes involved in p53 signalling pathway (cell cycle arrest), IL-10 (anti-inflammatory response), regulation of apoptosis, cell death, RNA transport and localization. This indicates that at this time point cells have proliferated sufficiently and are beginning to differentiate. At 48 hours after wounding, we observed mainly up regulation of miRNA genes responsible for silencing genes involved in protein catabolic processes, alternative splicing, spectrins, mRNA splicing and processing as well as methylation indicating that cells are undergoing physiological processes and restoring a normal phenotype.

## Discussion

The main finding of this study is the involvement of multiple miRNA genes in the process of epithelial wound repair *in vitro*. We found three distinct expression patterns of miRNA genes clusters that are predicted to further regulate numerous pathways and biological processes involved in wound repair. We have applied here for the first time the cluster analysis of time-series miRNA expression data (using STEM) to identify basic patterns and predict pathways (using DAVID) involved in repair processes of airway epithelium.

Such an approach has enabled us to identify common miRNA expression profiles during wound repair giving comprehensive information about activated miRNA genes. The relationships amongst these genes, their regulation and coordination during wound repair over time can also be explored. Further validation of individual protein, gene or miRNA changes will be required in subsequent studies, but it seems clear that specific expression profiles of clusters of miRNAs correlates with repair of mechanically induced damage to the epithelium. For expression profile 16 we demonstrated that, among other plausible signalling pathways, the neurotrophin signalling pathway may be involved in wound repair in epithelial cells, in addition to the inflammatory response in airway epithelium in allergy and asthma as reported previously [35-38]. The involvement in wound repair may further suggest that this pathway is important in the regulation of airway remodelling in asthma. Indeed, in the study by Kilic et al. [39] it was observed that blocking one of the neurotrophins, nerve growth factor (NGF), prevented subepithelial fibrosis in a mouse model of asthma and that NGF overexpression exerted a direct effect on collagen expression in murine lung fibroblasts. The involvement of neurotrophins in repair processes has been also confirmed recently by Palazzo et al. [40] in wound healing in dermal fibroblasts. Moreover, miRNAs involved in this pathway such as the miR-200 family were reported to control epithelial-mesenchymal transition (EMT) [41], the process that is suggested to underlie airway remodelling in asthma. In the recent study of Ogawa et al. [42] utilising a mouse model of asthma, it was observed that mice challenged with house dust mite allergen exhibited an increase in NGF that was primarily expressed in bronchial epithelium and was positively correlated with airway hyperresponsiveness and substance P-positive nerve fibers. However in this model siRNA targeted NGF inhibited hyperresponsiveness and modulation of innervation but not subepithelial fibrosis and allergic inflammation.

For expression profile 1 we observed a significant down-regulation at the beginning of wound repair followed by sharp increase in miRNA expression with a maximum at 16 hours after cell damage. This may indicate the induction of the six pathways predicted by enrichment analysis in the early phase of wound repair, which are then being switched off by the miRNAs with increased expression up to 16 hours post-wounding.

The process of wound repair *in vivo* in the airways involves cell spreading and migration as the primary mechanisms in the first 12–24 hours after injury, while proliferation begins by 15–24 h and continues for days to weeks. Similarly in our study we have confirmed that epithelial wound repair *in vitro* mimics the *in vivo* situation but in a shorter time frame, and that in its early stage this involves spreading and migration of neighbouring epithelial cells to cover the damaged area (2 and 4 hours after wounding). This is followed by migration and proliferation of progenitor cells to restore cell numbers (8 and 16 hours after cell damage) and differentiation to restore function (24 and 48 hours post-wounding) (Figure 1) [43-48].

Analysis of miRNAs involved at only specific time points of wound repair revealed that during the early stages numerous miRNAs are being significantly up-regulated, switching off pathways regulating cell proliferation and differentiation and activating cellular stress responses (chemokine signalling pathway) as well as cell migration and cell death (corresponding to time points at 2, 4 and 8 hours after injury). Furthermore, at later time points cells are undergoing intensive proliferation and secreting extracellular matrix which is supported by the involvement of ERBB signalling pathway and NFAT pathway stimulating cell proliferation and the regulation of transcription of immune genes (that corresponds to 16 hours after injury). Once confluent, cells restore their phenotype so that the cell cycle is arrested (inhibition of cell division) and differentiation processes are switched on. In parallel to this, the IL-10 anti-inflammatory signalling pathway is induced to deactivate immune cells stimulated during the early stages of wound repair.

## Conclusions

In summary, we report here for the first time that expression of multiple miRNAs is significantly altered during airway epithelium wound repair processes. Different patterns of expression have been observed and the target genes of those miRNA clusters coordinate several biological pathways involved in the repair of injury. Our work provides a starting point for a systematic analysis of mRNA targets specific for wound repair. This will help to identify regulatory networks controlling these processes in airway epithelium to better understand their involvement in respiratory diseases.

## Competing interests

The authors declare that they have no competing interests.

## Authors’ contributions

AS performed in vitro cell experiments and wounding assays, miRNA profiling, data analysis, cluster and pathway analysis, drafted the paper and approved its final version. PL contributed to the study design and methodology regarding cell experiments drafted the paper and approved its final version. JWH contributed to the study design and methodology regarding miRNA analysis, drafted the paper and approved its final version.

## Pre-publication history

The pre-publication history for this paper can be accessed here:

http://www.biomedcentral.com/1471-2466/13/63/prepub

## Supplementary Material

Additional file 1 **List of significantly modulated mature miRNAs (>10.0-fold) and their respective fold induction at each time point.** * miRNAs with significant change in expression at one time point only (marked in red). Click here for file

Additional file 2 Fold change of the top ten miRNAs undergoing significant modulation (>10-fold) during wound repair process at, at least, five time points. Click here for file

Additional file 3 MiRNA genes assigned to each expression profile during wound repair (values given for each time point represent expression change after normalization in STEM software). Click here for file

Additional file 4 The significantly overrepresented pathways (enriched) in the analysed sets of target genes of miRNAs included in the profile 16. Click here for file

Additional file 5 The significantly overrepresented pathways (enriched) in the analysed sets of target genes of miRNAs included in the profile 1. Click here for file

Additional file 6 The most significant biological processes predicted using DAVID tool undergoing regulation of miRNA target genes from the same expression profile (processes were ranked based on their Fisher Exact Probability value from the gene enrichment analysis to identify those showing significant overrepresentation). Click here for file

Additional file 7 Neurotrophin signaling pathway with miRNA genes and their predicted targets. Click here for file
