# Supplementary material for: Altered microRNA expression profile during epithelial wound repair in bronchial epithelial cells
Source: BMC Pulm Med. 2013 Nov 5;13:63. doi: 10.1186/1471-2466-13-63 (PMC4229315; doi:10.1186/1471-2466-13-63)
Supplement: Additional file 1 — List of significantly modulated mature miRNAs (>10.0-fold) and their respective fold induction at each time point. * miRNAs with significant change in expression at one time point only (marked in red). [file 1471-2466-13-63-S1.docx]

Additional file 1. List of significantly modulated mature miRNAs (>10.0-fold) and their respective fold induction at each time point

* miRNAs with significant change in expression at one time point only (marked in red)

| Upregulation | Fold change | Downregulation | Fold change |
| --- | --- | --- | --- |
| **2 hrs after wounding** | | | |
| hsa-miR-1243-002854 | 604.2724 | hsa-miR-188-3p-002106 | 0.0008 |
| hsa-miR-609-001573 | 79.5424 | hsa-miR-888-002212 | 0.0022 |
| hsa-miR-411-001610 | 43.1819 | hsa-miR-342-5p-002147 | 0.0118 |
| mmu-miR-137-001129 | 37.3266 | hsa-miR-330-5p-002230 | 0.0133 |
| hsa-let-7b#-002404 | 36.583 | hsa-miR-891b-002210 | 0.0242 |
| hsa-miR-629-002436 | 36.4477 | hsa-miR-331-5p-002233 | 0.0299 |
| hsa-miR-384-000574 | 25.1454 | hsa-miR-576-5p-002350 | 0.0328 |
| hsa-miR-483-5p-002338 | 21.7845 | hsa-miR-641-001585 | 0.0412 |
| hsa-miR-328-000543 | 19.0398 | hsa-miR-582-3p-002399 | 0.046 |
| hsa-miR-489-002358 | 17.4877 | hsa-miR-362-3p-002117 | 0.0686 |
| hsa-miR-518b-001156 | 17.3355 | hsa-miR-592-001546 | 0.0799 |
| mmu-miR-495-001663 | 16.8663 | hsa-miR-516a-5p-002416 | 0.0809 |
| hsa-miR-193a-3p-002250 | 16.5583 |  |  |
| hsa-miR-642-001592 | 13.3619 |  |  |
| hsa-miR-520c-3p-002400 | 12.7061 |  |  |
| hsa-miR-147b-002262 | 10.9207 |  |  |
| hsa-miR-542-5p-002240 | 10.7757 |  |  |
| **4 hrs after wounding** | | | |
| hsa-miR-609-001573 | 312.0607 | hsa-miR-188-3p-002106 | 0.0007 |
| mmu-miR-137-001129 | 201.2323 | hsa-miR-342-5p-002147 | 0.002 |
| hsa-miR-107-000443 | 118.3338 | hsa-miR-576-5p-002350 | 0.0043 |
| hsa-miR-411-001610 | 111.1598 | hsa-miR-888-002212 | 0.0057 |
| hsa-miR-193a-3p-002250 | 75.7727 | hsa-miR-885-3p-002372 | 0.0103 |
| hsa-miR-150-000473 | 73.986 | hsa-miR-891b-002210 | 0.0214 |
| hsa-miR-147b-002262 | 65.5334 | hsa-miR-24-1#-002440 | 0.0385 |
| hsa-miR-328-000543 | 41.6189 | hsa-miR-516-3p-001149 | 0.0548 |
| hsa-miR-219-1-3p-002095 | 36.4937 | hsa-miR-376b-001102 | 0.0598 |
| hsa-miR-302b-000531 | 20.0886 | hsa-miR-26a-2#-002115 | 0.0705 |
| hsa-miR-489-002358 | 19.5574 | hsa-miR-516a-5p-002416 | 0.0716 |
| hsa-miR-22-000398 | 15.5766 | hsa-miR-502-001109 | 0.0728 |
| hsa-miR-542-5p-002240 | 14.5463 | hsa-miR-19a#-002424 | 0.0806 |
| hsa-miR-518e-002395 | 14.5015 | hsa-miR-432#-001027 | 0.0915 |
| hsa-miR-582-5p-001983 | 14.1639 |  |  |
| mmu-miR-134-001186 | 13.8798 |  |  |
| hsa-miR-889-002202 | 11.7223 |  |  |
| hsa-miR-520D-3P-002743 | 10.6802 |  |  |
| hsa-miR-455-3p-002244 | 10.5988 |  |  |
| hsa-miR-502-3p-002083 | 10.2315 |  |  |
| hsa-miR-483-5p-002338 | 10.1177 |  |  |
| **8 hrs after wounding** | | | |
| hsa-miR-609-001573 | 3497.2458 | hsa-miR-342-5p-002147 | 0.0015 |
| mmu-miR-137-001129 | 357.444 | hsa-miR-188-3p-002106 | 0.0087 |
| mmu-miR-495-001663 | 83.3585 | hsa-miR-450b-5p-002207 | 0.012 |
| hsa-miR-193a-3p-002250 | 72.6988 | hsa-miR-572-001614 | 0.0161 |
| hsa-miR-411-001610 | 53.9956 | hsa-miR-576-5p-002350 | 0.0244 |
| hsa-miR-525-3p-002385 | 50.3488 | hsa-miR-582-3p-002399 | 0.0306 |
| hsa-miR-1249-002868 | 44.7812 | hsa-miR-24-1#-002440 | 0.0364 |
| mmu-miR-129-3p-001184 | 43.1188 | hsa-miR-376b-001102 | 0.045 |
| hsa-miR-886-3p-002194 | 39.0165 | hsa-miR-516a-5p-002416 | 0.0538 |
| hsa-miR-520c-3p-002400 | 34.4473 | hsa-miR-516-3p-001149 | 0.0654 |
| mmu-miR-615-001960 | 32.9299 | hsa-miR-1300-002902 | 0.0712 |
| hsa-miR-328-000543 | 31.8307 | hsa-miR-500-001046 | 0.0843 |
| hsa-miR-147b-002262 | 27.2193 | hsa-miR-449-001030 | 0.089 |
| hsa-miR-139-3p-002313 | 27.0462 | hsa-miR-542-3p-001284 | 0.0925 |
| mmu-miR-134-001186 | 26.5965 |  |  |
| hsa-miR-518b-001156 | 25.4355 |  |  |
| hsa-miR-219-1-3p-002095 | 24.2423 |  |  |
| hsa-miR-373-000561 | 24.1396 |  |  |
| hsa-miR-147-000469 | 23.037 |  |  |
| hsa-miR-216a-002220 | 15.5252 |  |  |
| hsa-miR-150-000473 | 15.4279 |  |  |
| hsa-miR-410-001274 | 12.6521 |  |  |
| hsa-miR-32-002109 | 11.4583 |  |  |
| hsa-miR-520D-3P-002743 | 11.349 |  |  |
| **16 hrs after wounding** | | | |
| hsa-miR-609-001573 | 3138.7785 | hsa-miR-188-3p-002106 | 0.0004 |
| mmu-miR-137-001129 | 175.9993 | hsa-miR-888-002212 | 0.0012 |
| hsa-miR-411-001610 | 82.7975 | hsa-miR-342-5p-002147 | 0.0012 |
| hsa-miR-139-3p-002313 | 51.1619 | hsa-miR-576-5p-002350 | 0.0026 |
| hsa-miR-193a-3p-002250 | 43.8721 | hsa-miR-597-001551 | 0.0079 |
| hsa-miR-328-000543 | 18.3341 | hsa-miR-891b-002210 | 0.0129 |
| hsa-miR-142-3p-000464 | 14.9275 | hsa-miR-204-000508 | 0.0143 |
| hsa-miR-487b-001285 | 13.4607 | hsa-miR-643-001594 | 0.0242 |
| hsa-miR-502-3p-002083 | 10.1851 | hsa-miR-582-3p-002399 | 0.0245 |
|  |  | hsa-miR-100#-002142 | 0.0316 |
|  |  | hsa-let-7c-000379 | 0.035 |
|  |  | hsa-miR-1300-002902 | 0.0467 |
|  |  | hsa-miR-545-002267 | 0.0479 |
|  |  | dme-miR-7-000268 | 0.0484 |
|  |  | hsa-miR-592-001546 | 0.0554 |
|  |  | mmu-miR-124a-001182 | 0.0568 |
|  |  | hsa-let-7b-002619 | 0.0609 |
|  |  | hsa-miR-371-3p-002124 | 0.0774 |
|  |  | hsa-miR-331-5p-002233 | 0.0846 |
|  |  | hsa-miR-521-001122 | 0.0866 |
|  |  | hsa-miR-374a#-002125 | 0.087 |
|  |  | hsa-miR-1243-002854 | 0.0872 |
|  |  | hsa-miR-605-001568 | 0.0883 |
|  |  | hsa-miR-520c-3p-002400 | 0.0929 |
|  |  | hsa-miR-330-5p-002230 | 0.095 |
|  |  | hsa-miR-450a-002303 | 0.0973 |
|  |  | hsa-miR-10b-002218 | 0.0975 |
| **24 hrs after wounding** | | | |
| hsa-miR-411-001610 | 63.1153 | hsa-miR-888-002212 | 0.0001 |
| mmu-miR-134-001186 | 18.4869 | hsa-miR-188-3p-002106 | 0.0005 |
| hsa-miR-139-3p-002313 | 17.2328 | hsa-miR-342-5p-002147 | 0.0013 |
| hsa-miR-143-002249 | 16.3833 | hsa-miR-576-5p-002350 | 0.0028 |
| hsa-miR-328-000543 | 16.1802 | hsa-miR-362-3p-002117 | 0.0055 |
| hsa-miR-485-3p-001277 | 15.6506 | hsa-miR-885-3p-002372 | 0.0066 |
| hsa-miR-142-3p-000464 | 14.6833 | hsa-miR-330-5p-002230 | 0.0076 |
| mmu-miR-137-001129 | 13.6204 | hsa-miR-597-001551 | 0.0085 |
| hsa-miR-542-5p-002240 | 12.0145 | hsa-miR-891b-002210 | 0.0138 |
|  |  | hsa-miR-545-002267 | 0.0255 |
|  |  | hsa-miR-582-3p-002399 | 0.0262 |
|  |  | hsa-miR-24-1#-002440 | 0.0419 |
|  |  | hsa-miR-516a-5p-002416 | 0.0462 |
|  |  | hsa-miR-192#-002272 | 0.0535 |
|  |  | hsa-let-7c-000379 | 0.0583 |
|  |  | hsa-miR-572-001614 | 0.0602 |
|  |  | hsa-miR-638-001582 | 0.0659 |
|  |  | hsa-miR-622-001553 | 0.0664 |
|  |  | hsa-let-7b-002619 | 0.078 |
|  |  | hsa-miR-149#-002164 | 0.0905 |
|  |  | hsa-miR-521-001122 | 0.0928 |
| **48 hrs after wounding** | | | |
| hsa-miR-609-001573 | 4781.6379 | hsa-miR-135b#-002159 | 0.016 |
| hsa-miR-520c-3p-002400 | 1211.632 | hsa-miR-891b-002210 | 0.0291 |
| hsa-miR-1249-002868 | 620.6651 | hsa-miR-885-3p-002372 | 0.0441 |
| hsa-miR-1291-002838 | 352.2416 | hsa-miR-582-3p-002399 | 0.0551 |
| hsa-miR-150-000473 | 234.1771 | hsa-miR-221#-002096 | 0.0742 |
| mmu-miR-137-001129 | 173.1145 | hsa-miR-376b-001102 | 0.0811 |
| hsa-miR-147b-002262 | 154.5396 | hsa-miR-576-5p-002350 | 0.0839 |
| mmu-miR-615-001960 | 137.8024 | hsa-miR-545-002267 | 0.0946 |
| hsa-miR-369-3p-000557 | 121.2427 | hsa-miR-188-3p-002106 | 0.0949 |
| hsa-miR-411-001610 | 117.3975 | hsa-miR-516a-5p-002416 | 0.097 |
| mmu-miR-379-001138 | 110.4951 | hsa-miR-888-002212 | 0.1005 |
| hsa-miR-328-000543 | 91.7739 | hsa-miR-616-002414 | 0.1064 |
| hsa-miR-452#-002330 | 80.4914 |  |  |
| hsa-miR-487a-001279 | 76.6061 |  |  |
| hsa-miR-875-5p-002203 | 76.3348 |  |  |
| hsa-miR-519e-002370 | 75.869 |  |  |
| hsa-miR-455-3p-002244 | 60.2198 |  |  |
| hsa-miR-142-3p-000464 | 46.8399 |  |  |
| hsa-miR-1243-002854 | 44.9803 |  |  |
| hsa-let-7b#-002404 | 44.5731 |  |  |
| hsa-miR-92a-1#-002137 | 43.3389 |  |  |
| hsa-miR-633-001574 | 42.8744 |  |  |
| hsa-miR-22-000398 | 32.5663 |  |  |
| hsa-miR-378-000567 | 31.9762 |  |  |
| hsa-miR-147-000469 | 30.497 |  |  |
| hsa-miR-107-000443 | 28.2808 |  |  |
| hsa-miR-489-002358 | 27.1962 |  |  |
| hsa-miR-582-5p-001983 | 26.8222 |  |  |
| hsa-miR-193a-3p-002250 | 23.8726 |  |  |
| hsa-miR-513-5p-002090 | 22.6412 |  |  |
| hsa-miR-548d-001605 | 21.7156 |  |  |
| hsa-miR-708-002341 | 20.3964 |  |  |
| hsa-miR-502-3p-002083 | 19.9094 |  |  |
| hsa-miR-149#-002164 | 19.26 |  |  |
| hsa-miR-101-002253 | 18.9113 |  |  |
| hsa-miR-1225-3P-002766 | 18.2682 |  |  |
| hsa-miR-584-001624 | 17.3918 |  |  |
| hsa-miR-29a#-002447 | 16.4589 |  |  |
| hsa-miR-320B-002844 | 16.3669 |  |  |
| hsa-miR-135a-000460 | 16.3189 |  |  |
| hsa-miR-219-1-3p-002095 | 16.0039 |  |  |
| hsa-miR-323-3p-002227 | 15.8348 |  |  |
| hsa-miR-744#-002325 | 14.3237 |  |  |
| hsa-miR-1275-002840 | 13.707 |  |  |
| hsa-miR-485-3p-001277 | 13.3888 |  |  |
| hsa-miR-155-002623 | 12.9041 |  |  |
| hsa-miR-642-001592 | 12.6602 |  |  |
| hsa-miR-548c-5p-002429 | 12.5154 |  |  |
| hsa-miR-570-002347 | 12.3931 |  |  |
| hsa-miR-23b-000400 | 11.7389 |  |  |
| mmu-miR-129-3p-001184 | 11.7184 |  |  |
| hsa-miR-652-002352 | 11.2796 |  |  |
| hsa-miR-34b-002102 | 11.1836 |  |  |
| hsa-miR-223-002295 | 11.0856 |  |  |
| hsa-miR-34a-000426 | 10.4461 |  |  |
| hsa-miR-202-002363 | 10.2762 |  |  |
| hsa-miR-1305-002867 | 10.2682 |  |  |
