# Supplementary material for: Altered microRNA expression profile during epithelial wound repair in bronchial epithelial cells
Source: BMC Pulm Med. 2013 Nov 5;13:63. doi: 10.1186/1471-2466-13-63 (PMC4229315; doi:10.1186/1471-2466-13-63)
Supplement: Additional file 2 — Fold change of the top ten miRNAs undergoing significant modulation (>10-fold) during wound repair process at, at least, five time points. [file 1471-2466-13-63-S2.docx]

Additional file 2. Fold change of the top ten miRNAs undergoing significant modulation (>10-fold) during wound repair process at, at least, five time points

| miRNA | baseline | 2 hrs | 4 hrs | 8 hrs | 16 hrs | 24 hrs | 48 hrs |
| --- | --- | --- | --- | --- | --- | --- | --- |
| **Upregulated** | | | | | | | |
| hsa-miR-609 | 1 | 79.5424 | 312.0607 | 3497.245 | 3138.778 | 0.406 | 4781.63 |
| hsa-miR-411 | 1 | 43.1819 | 111.1598 | 53.9956 | 82.7975 | 63.1153 | 117.397 |
| mmu-miR-137 | 1 | 37.3266 | 201.2323 | 357.444 | 175.9993 | 13.6204 | 173.114 |
| hsa-miR-328 | 1 | 19.0398 | 41.6189 | 31.8307 | 18.334 | 16.180 | 91.770 |
| hsa-miR-193a-3p | 1 | 16.5583 | 75.7727 | 72.6988 | 43.872 | 0.589 | 23.8726 |
| **Downregulated** | | | | | | | |
| hsa-miR-188-3p | 1 | 0.0008 | 0.0007 | 0.0087 | 0.0004 | 0.000 | 0.094 |
| hsa-miR-888 | 1 | 0.0022 | 0.0057 | 0.1201 | 0.0012 | 1.00E-04 | 0.100 |
| hsa-miR-342-5p | 1 | 0.0118 | 0.002 | 0.0015 | 0.0012 | 0.001 | 0.423 |
| hsa-miR-576-5p | 1 | 0.0328 | 0.0043 | 0.0244 | 0.0026 | 0.002 | 0.083 |
| hsa-miR-891b | 1 | 0.0242 | 0.0214 | 0.3202 | 0.0129 | 0.013 | 0.029 |
