# Supplementary material for: Altered microRNA expression profile during epithelial wound repair in bronchial epithelial cells
Source: BMC Pulm Med. 2013 Nov 5;13:63. doi: 10.1186/1471-2466-13-63 (PMC4229315; doi:10.1186/1471-2466-13-63)
Supplement: Additional file 3 — MiRNA genes assigned to each expression profile during wound repair (values given for each time point represent expression change after normalization in STEM software). [file 1471-2466-13-63-S3.docx]

Additional file 3. MiRNA genes assigned to each expression profile during wound repair (values given for each time point represent expression change after normalization in STEM software)

| **Gene** | **0 h** | **2 h** | **4 h** | **8 h** | **16 h** | **24 h** | **48 h** |
| --- | --- | --- | --- | --- | --- | --- | --- |
| **Profile 16 (P=3.6E-13)** | | | | | | | |
| hsa-miR-125A-5P-002198 | 0.0 | -0.10 | -0.02 | 0.30 | 0.29 | 0.43 | -0.65 |
| hsa-miR-183-002269 | 0.0 | -0.46 | -1.06 | -0.41 | 0.33 | 0.21 | -2.10 |
| hsa-miR-200A-000502 | 0.0 | -0.52 | -0.90 | -0.20 | 0.09 | 0.09 | -1.57 |
| hsa-miR-200B-002251 | 0.0 | -0.80 | -1.37 | -0.55 | -0.14 | -0.30 | -2.51 |
| hsa-miR-30B-000602 | 0.0 | -0.55 | -0.94 | 0.04 | 0.52 | 0.52 | -2.17 |
| hsa-miR-339-3P-002184 | 0.0 | -0.63 | -0.71 | -0.65 | -0.36 | -0.56 | -1.56 |
| hsa-miR-1260-002896 | 0.0 | 0.47 | 0.81 | 0.94 | 1.77 | 1.10 | -0.78 |
| hsa-miR-130B#-002114 | 0.0 | 0.51 | 0.64 | 0.81 | 1.86 | 1.41 | -1.31 |
| hsa-miR-151-3P-002254 | 0.0 | 0.37 | 0.73 | 1.02 | 1.51 | 0.89 | -0.70 |
| hsa-miR-151-5P-002642 | 0.0 | 0.59 | 0.65 | 1.26 | 1.62 | 1.06 | -1.36 |
| hsa-miR-181A-2#-002317 | 0.0 | 0.07 | 0.47 | 0.76 | 1.38 | 0.87 | -1.13 |
| hsa-miR-30A-3P-000416 | 0.0 | 0.38 | 0.51 | 0.99 | 1.22 | 0.81 | -1.02 |
| hsa-miR-30D-000420 | 0.0 | -0.09 | 0.32 | 0.84 | 1.77 | 1.25 | -1.28 |
| hsa-miR-30E-3P-000422 | 0.0 | 0.40 | 0.73 | 1.03 | 1.47 | 1.09 | -1.04 |
| hsa-miR-550-002410 | 0.0 | 0.42 | 0.60 | 0.53 | 1.19 | 0.60 | -0.90 |
| hsa-miR-935-002178 | 0.0 | 0.63 | 0.45 | 0.44 | 1.19 | 0.64 | -0.68 |
| hsa-miR-941-002183 | 0.0 | 0.54 | 0.28 | 0.93 | 2.20 | 0.36 | -0.91 |
| **Profile 1** **(P=3.7E-11)** | | | | | | | |
| hsa-miR-149-002255 | 0.00 | -0.90 | -1.12 | -0.59 | -0.05 | -0.11 | -1.43 |
| hsa-miR-15B-000390 | 0.00 | -0.55 | -1.13 | -0.06 | 0.62 | 0.48 | -1.60 |
| hsa-miR-195-000494 | 0.00 | -0.75 | -1.26 | -0.17 | 0.02 | -0.18 | -1.82 |
| hsa-miR-218-000521 | 0.00 | -0.48 | -0.82 | -0.01 | 0.30 | 0.28 | -1.25 |
| hsa-miR-222-002276 | 0.00 | -0.51 | -0.58 | 0.23 | 0.67 | 0.55 | -1.11 |
| hsa-miR-331-000545 | 0.00 | -0.32 | -0.68 | -0.09 | 0.45 | 0.38 | -1.09 |
| hsa-miR-374-000563 | 0.00 | -0.24 | -0.48 | 0.35 | 0.99 | 0.87 | -0.99 |
| hsa-miR-425-5P-001516 | 0.00 | -0.22 | -0.83 | 0.13 | 0.42 | 0.43 | -1.11 |
| hsa-miR-574-3P-002349 | 0.00 | -0.37 | -0.93 | -0.29 | 0.26 | 0.10 | -1.42 |
| hsa-miR-590-5P-001984 | 0.00 | -0.73 | -1.23 | -0.49 | -0.34 | -0.33 | -1.75 |
| hsa-miR-9-000583 | 0.00 | -0.91 | -1.53 | -0.50 | -0.10 | -0.21 | -1.84 |
| mmu-miR-374-5P-001319 | 0.00 | -0.38 | -0.66 | 0.29 | 0.71 | 0.54 | -1.13 |
| **Profile 18** **(P=7.8E-5)** | | | | | | | |
| hsa-miR-197-000497 | 0.00 | -0.60 | -0.48 | 0.35 | 0.96 | 0.69 | -0.72 |
| hsa-miR-532-3P-002355 | 0.00 | -0.57 | -0.40 | 0.35 | 0.80 | 0.98 | -1.30 |
| hsa-miR-200A#-001011 | 0.00 | -0.16 | 0.61 | 0.87 | 1.12 | 1.10 | -0.24 |
| hsa-miR-30A-5P-000417 | 0.00 | 0.58 | 0.98 | 1.46 | 2.46 | 1.73 | -0.60 |
| hsa-miR-34A#-002316 | 0.00 | 0.57 | 0.85 | 1.22 | 1.94 | 1.39 | -0.51 |
| hsa-miR-664-002897 | 0.00 | 0.54 | 1.15 | 1.54 | 2.09 | 1.71 | 0.21 |
| hsa-miR-93#-002139 | 0.00 | 0.57 | 1.12 | 1.42 | 1.90 | 1.46 | -0.20 |
