# Supplementary figures and images for: Altered microRNA expression profile during epithelial wound repair in bronchial epithelial cells
Source: BMC Pulm Med. 2013 Nov 5;13:63. doi: 10.1186/1471-2466-13-63 (PMC4229315; doi:10.1186/1471-2466-13-63)

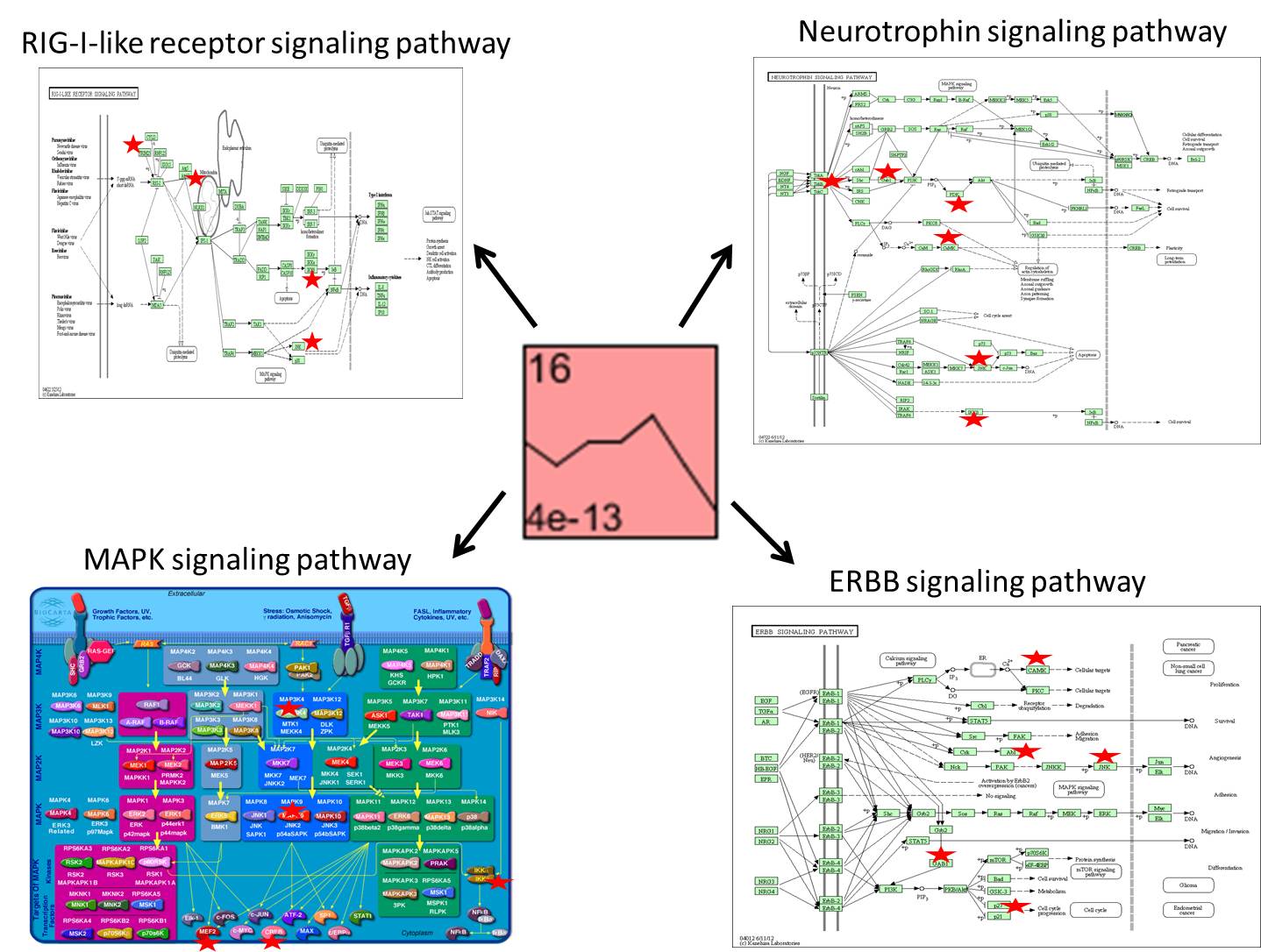

Supplement: Additional file 4 — The significantly overrepresented pathways (enriched) in the analysed sets of target genes of miRNAs included in the profile 16. [file 1471-2466-13-63-S4.jpeg]

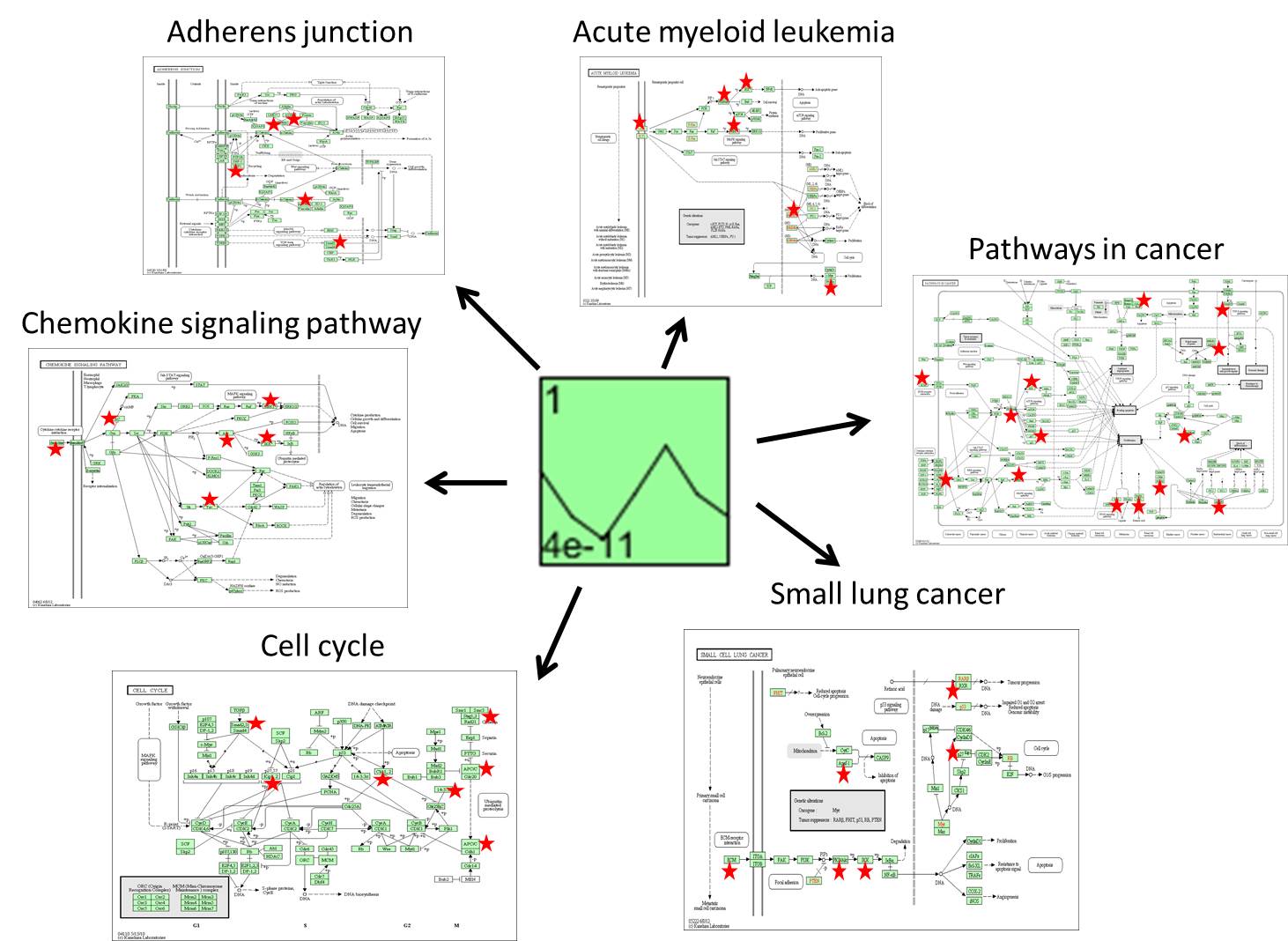

Supplement: Additional file 5 — The significantly overrepresented pathways (enriched) in the analysed sets of target genes of miRNAs included in the profile 1. [file 1471-2466-13-63-S5.jpeg]

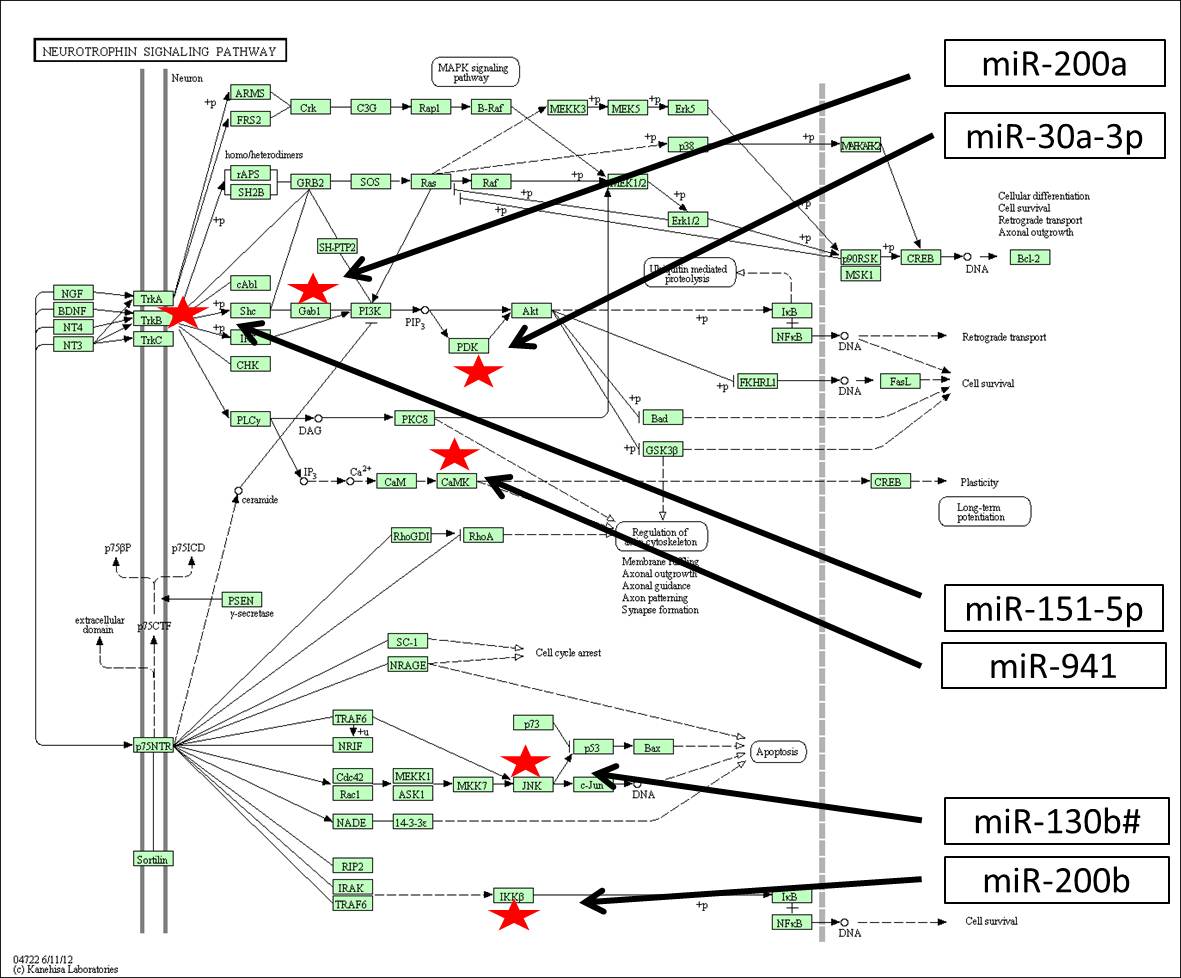

Supplement: Additional file 7 — Neurotrophin signaling pathway with miRNA genes and their predicted targets. [file 1471-2466-13-63-S7.jpeg]
