# Supplementary material for: Altered microRNA expression profile during epithelial wound repair in bronchial epithelial cells
Source: BMC Pulm Med. 2013 Nov 5;13:63. doi: 10.1186/1471-2466-13-63 (PMC4229315; doi:10.1186/1471-2466-13-63)
Supplement: Additional file 6 — The most significant biological processes predicted using DAVID tool undergoing regulation of miRNA target genes from the same expression profile (processes were ranked based on their Fisher Exact Probability value from the gene enrichment analysis to identify those showing significant overrepresentation). [file 1471-2466-13-63-S6.docx]

Additional file 6. The most significant biological processes predicted using DAVID tool undergoing regulation of miRNA target genes from the same expression profile (processes were ranked based on their Fisher Exact Probability value from the gene enrichment analysis to identify those showing significant overrepresentation).

| Biological process | Gene count | P-value | Genes |
| --- | --- | --- | --- |
| **Profile 16** | | | |
| developmental protein | 24 | 1.067E-4 | CHURC1, NOG, MEF2A, ARX, NDE1, DIP2A, HAND1, AGGF1, B3GNT5, SEMA3E, FBXW4, RHOB, UNC5C, DCX, LFNG, DAZ3, DAZ4, DAZ1, DAZ2, EMX2, DPYSL2, SNAI1, FXR1, NTRK2, LRP6, NHLH2, SEMA4D |
| phosphoprotein | 118 | 2.030E-4 | ENAH, MEF2A, IL16, ZNRF1, ACBD3, MAP3K4, MFHAS1, AAK1, TARDBP, GAB1, PELI2, ASPH, UNC5C, PAG1, POLH, KIAA1804, UBE2J1, MECP2, HMG20A, COLEC12, FAM104A, PPARGC1B, SPAG9, PGM3, RNF138, MAPK9, UNC13C, ZNHIT6, UGGT1, SDAD1, DBNDD2, ENPP1, SNRPB2, SOX5, MAPKAPK3, DUSP10, AKAP12, AKAP11, TCERG1, AGGF1, SYAP1, TFDP2, CLASP2, NOVA1, DNMT3A, GABARAPL2, EPB41, OSBPL8, LPP, PTPN4, CCDC80, PTPN13, REEP1, KCTD5, ARMC1, DOCK4, FXR1, CDKN1B, NTRK2, ANTXR2, IKBKB, ABL2, ZEB1, PDCD4, CDT1, NDE1, GPKOW, CTAGE1, RHOB, FAM83F, SEC23A, TBL1XR1, ACTA1, SCAI, PKN2, TTF1, GLCCI1, FOXJ3, RFTN1, ZCCHC14, EIF4G2, MYRIP, SBF1, RRM2, AVEN, CPSF6, SEMA4D, USP25, CAMK1D, MFSD6, SBNO1, CAMK2G, SNX16, CDC73, FKBP1A, NFYA, NUFIP2, STAU1, HAND1, BCL11B, PKD2, DCX, TNRC6B, NEFL, GDI1, BRD1, CREB1, FRMPD1, ELAVL1, BIRC6, TRIM25, DPYSL2, SNAI1, CDC25A, RAB32, KCMF1, GFPT1, FAM126A |
| alternative splicing | 120 | 3.140E-4 | ENAH, MEF2A, SCN3A, IL16, DNAJB14, ZNRF1, FAHD1, KLHL5, MAP3K4, KCNK6, FAP, GAB1, UNC5C, FBXO22, POLH, KIAA1804, MECP2, HMG20A, COLEC12, ICA1L, PTPDC1, PPARGC1B, SPAG9, ANKRD13C, MAPK9, RNF138, ASB3, PIAS2, UGGT1, CLCN3, SDAD1, C11ORF63, DBNDD2, SOX5, DUSP10, AKAP12, FAM19A2, ZPLD1, SULT4A1, SYPL1, TCERG1, AGGF1, TTBK1, TFDP2, CASZ1, CLASP2, LFNG, NOVA1, EPB41, OSBPL8, C17ORF101, CCDC80, PTPN13, DOCK4, FXR1, FAM198B, DIO2, NTRK2, NLGN4X, ANTXR2, SCN8A, ABL2, SEL1L, C1ORF38, CHURC1, LRRC17, KCNK10, NDE1, DIP2A, FAM115A, PRMT8, ZFYVE9, CTAGE1, JAKMIP2, FAM83F, KIAA1324L, ZDHHC3, NOL3, SCAI, CNPY2, CCDC47, PCDH9, FOXJ3, ZCCHC14, EIF4G2, SS18, DNAJC27, SBF1, SLC26A7, FAM120C, CLIC5, CPSF6, USP25, CAMK1D, SUPT3H, SBNO1, APH1A, C10ORF46, ATG12, CAMK2G, NFYA, PRDM16, STAU1, BCL11B, ADAT2, DCX, TNRC6B, MTMR7, DAZ3, DAZ4, DAZ1, DAZ2, NUB1, NXNL2, CREB1, CEP63, CDC25A, RASSF6, PRLR, GFPT1, FAM126A, ALKBH4, TP53INP1 |
| splice variant | 120 | 3.497E-4 | ENAH, MEF2A, SCN3A, IL16, DNAJB14, ZNRF1, FAHD1, KLHL5, MAP3K4, KCNK6, FAP, GAB1, UNC5C, FBXO22, POLH, KIAA1804, MECP2, HMG20A, COLEC12, ICA1L, PTPDC1, PPARGC1B, SPAG9, ANKRD13C, MAPK9, RNF138, ASB3, PIAS2, UGGT1, CLCN3, SDAD1, C11ORF63, DBNDD2, SOX5, DUSP10, AKAP12, FAM19A2, ZPLD1, SULT4A1, TCERG1, SYPL1, AGGF1, TTBK1, TFDP2, CASZ1, CLASP2, LFNG, NOVA1, DNMT3A, EPB41, OSBPL8, C17ORF101, CCDC80, PTPN13, DOCK4, FXR1, FAM198B, DIO2, NTRK2, NLGN4X, ANTXR2, SCN8A, ABL2, SEL1L, C1ORF38, CHURC1, LRRC17, KCNK10, DIP2A, NDE1, FAM115A, PRMT8, ZFYVE9, JAKMIP2, FAM83F, KIAA1324L, ZDHHC3, NOL3, SCAI, CNPY2, CCDC47, PCDH9, FOXJ3, ZCCHC14, EIF4G2, SS18, DNAJC27, SBF1, SLC26A7, FAM120C, CLIC5, CPSF6, USP25, CAMK1D, SUPT3H, SBNO1, APH1A, C10ORF46, ATG12, CAMK2G, NFYA, PRDM16, STAU1, BCL11B, ADAT2, DCX, TNRC6B, MTMR7, DAZ3, DAZ4, DAZ1, DAZ2, NUB1, NXNL2, CREB1, CEP63, CDC25A, RASSF6, PRLR, GFPT1, FAM126A, ALKBH4, TP53INP1 |
| phosphate metabolic process | 28 | 4.674E-4 | ENPP1, CAMK2G, STYX, DUSP10, MAPKAPK3, MAP3K4, TTBK1, AAK1, GAB1, MTMR7, PDK1, KIAA1804, CREB1, PTPN4, PKN2, PTPN13, PTPDC1, CDC25A, SPAG9, PRLR, DUSP1, SBF1, NTRK2, MAPK9, ATP6V0A4, IKBKB, ABL2, CAMK1D |
| phosphorus metabolic process | 28 | 4.674E-4 | ENPP1, CAMK2G, STYX, DUSP10, MAPKAPK3, MAP3K4, TTBK1, AAK1, GAB1, MTMR7, PDK1, KIAA1804, CREB1, PTPN4, PKN2, PTPN13, PTPDC1, CDC25A, SPAG9, PRLR, DUSP1, SBF1, NTRK2, MAPK9, ATP6V0A4, IKBKB, ABL2, CAMK1D |
| mutagenesis site | 42 | 0.001 | CLCN3, MEF2A, SCN3A, IL16, GNPNAT1, MAPKAPK3, ZNRF1, CDT1, MAP3K4, TCERG1, CISD1, PRMT8, GPR26, KCNK6, ZFYVE9, TARDBP, PKD2, RALB, RHOB, PAG1, DNMT3A, POLH, EPB41, NUB1, LPP, PCTP, UBE2J1, UBE2I, TRIM25, DPYSL2, RFTN1, CDC25A, PPARGC1B, PGM3, RAB32, CDKN1B, RRM2, CPSF6, PIAS2, IKBKB, UGGT1, CAMK1D |
| intracellular signaling cascade | 30 | 0.004 | ADCY1, DUSP10, MAPKAPK3, AKAP11, MAP3K4, GALR1, GAB1, RALB, PKD2, RHOB, DCX, PAG1, PDK1, GDI1, KLF9, KIAA1804, COLEC12, CEP63, PPARGC1B, SS18, RAB32, SPAG9, DNAJC27, PRLR, DUSP1, MAPK9, PIAS2, ASB3, UNC13C, IKBKB |
| intracellular organelle lumen | 33 | 0.035 | SUPT3H, SDAD1, CDC73, ZEB1, NFYA, PRDM16, CDT1, GPKOW, HAND1, TARDBP, TFDP2, NOVA1, ACSM2B, PDK1, DNMT3A, BRD1, TBL1XR1, NOL3, POLH, CREB1, ACSM2A, HMG20A, TTF1, UBE2I, TRIM25, PPARGC1B, CDC25A, FXR1, CPSF6, PIAS2, AARS2, UGGT1, CROT |
| transcription regulator activity | 30 | 0.035 | SUPT3H, MEF2A, CHURC1, SOX5, ZEB1, NFYA, PRDM16, ARX, TCERG1, HAND1, TARDBP, TFDP2, PLAGL2, TBL1XR1, KLF9, CREB1, EMX2, SCAI, MECP2, TTF1, HMG20A, TRIM25, UBE2I, FOXJ3, PPARGC1B, ZNF138, BTG1, NHLH2, PIAS2, IKBKB |
| transferase | 26 | 0.039 | GNPNAT1, CAMK2G, MAPKAPK3, SULT4A1, MAP3K4, B3GNT5, PRMT8, TTBK1, AAK1, B4GALT6, LFNG, PDK1, DNMT3A, MGAT4A, ZDHHC3, POLH, KIAA1804, PKN2, GFPT1, NTRK2, MAPK9, IKBKB, UGGT1, ABL2, CROT, CAMK1D |
| cytoplasm | 53 | 0.041 | ENAH, EIF2C1, IL16, PDCD4, NDE1, KLHL5, ZFYVE9, NOL3, ACTA1, PCTP, PKN2, SCAI, SPAG9, MYRIP, RRM2, CLIC5, UNC13C, CAMK1D, ATG12, AKAP12, DUSP10, MAPKAPK3, FAM19A2, FKBP1A, AKAP11, NUFIP2, STAU1, SULT4A1, AGGF1, TTBK1, CLASP2, TNRC6B, DCX, MTMR7, DAZ3, DNMT3A, GDI1, RANBP17, DAZ4, DAZ1, DAZ2, EPB41, LPP, PTPN4, PTPN13, FRMPD1, ELAVL1, TRIM25, DPYSL2, CEP63, KCTD5, FXR1, CDKN1B, IKBKB, ABL2, FAM126A |
| organelle lumen | 33 | 0.046 | SUPT3H, SDAD1, CDC73, ZEB1, NFYA, PRDM16, CDT1, GPKOW, HAND1, TARDBP, TFDP2, NOVA1, ACSM2B, PDK1, DNMT3A, BRD1, TBL1XR1, NOL3, POLH, CREB1, ACSM2A, HMG20A, TTF1, UBE2I, TRIM25, PPARGC1B, CDC25A, FXR1, CPSF6, PIAS2, AARS2, UGGT1, CROT |
| **Profile 1** | | | |
| phosphoprotein | 119 | 8.934E-9 | MPZL1, SLC9A6, IL16, CTPS, RBM6, ENPEP, CUL2, GATA3, SLC4A1, STAG2, PTPRJ, RSBN1L, ACTN4, STRN3, ACTN1, BCL2L11, TESK2, VGLL4, CHORDC1, CD226, EIF2AK4, CRTC2, CHCHD3, AKAP12, CHEK1, EPHB3, PEX5L, MED12L, MIA3, NIPBL, SFRS16, KIAA1012, BAI1, DUSP16, SSX2IP, C11ORF58, GAD1, IQSEC2, ESRRA, MAP2K1, ACACA, CDC23, SPRYD3, SMAD2, SKI, VAV2, SFMBT1, CDKN1C, EPHA4, LYVE1, CDKN1B, SEMA6D, EPHA8, LASP1, SLAIN2, PARP8, HIVEP2, BET1L, SYTL2, IKBKB, UTP14C, AHDC1, FAM91A1, ELF2, ATP10B, ARHGAP19, PDIA6, FAM63B, SKAP1, SPRY1, ZFYVE16, AP3B2, CNTNAP1, USP16, AKT3, AP3B1, KHDRBS2, TRPM7, MESDC1, RUNX1T1, PFKP, TLE4, FOXJ3, HERC2, FLNC, MBD2, GRM1, ZNF335, SEMA4G, NCK1, KPNA3, KSR1, CLOCK, MUM1L1, MKNK1, KIT, CDH5, CHD7, ACSL1, WAC, KIF21A, FYCO1, BAHD1, UPF2, SHMT2, RBM24, CEBPB, DLGAP2, MSH2, LMNA, RACGAP1, ITPR1, C12ORF41, RGS14, YWHAG, SP1, APAF1, SETD2, DUSP8 |
| cell fraction | 26 | 3.044E-4 | SLC9A6, PEX5L, CDH5, ACSL1, STAC, BCHE, PEX13, PCSK6, PPAP2A, RECK, MAP2K1, KIF5A, LMNA, ACACA, PFKP, CYP26A1, GRM1, ITPR1, BCL2L11, LAMP1, CYP7B1, LYVE1, BACE1, SYTL2, APAF1, IKBKB |
| phosphorus metabolic process | 25 | 5.563E-4 | CCL2, STYX, MKNK1, CHEK1, KIT, EPHB3, ATP5G3, DUSP16, PPAP2A, AKT3, COX15, PTPRJ, ADAM10, MAP2K1, TRPM7, MSH2, SMAD2, GRM1, EPHA4, EPHA8, TESK2, KSR1, IKBKB, DUSP8, EIF2AK4 |
| phosphate metabolic process | 25 | 5.563E-4 | CCL2, STYX, MKNK1, CHEK1, KIT, EPHB3, ATP5G3, DUSP16, PPAP2A, AKT3, COX15, PTPRJ, ADAM10, MAP2K1, TRPM7, MSH2, SMAD2, GRM1, EPHA4, EPHA8, TESK2, KSR1, IKBKB, DUSP8, EIF2AK4 |
| mutagenesis site | 38 | 0.001 | CRTC2, MPZL1, CCL2, NDST1, IL16, MKNK1, CHEK1, KIT, SKAP1, CUL2, HTRA2, ZFYVE16, USP16, FGFBP1, AKT3, UPF2, ESRRA, MAP2K1, KIF5A, MSH2, TRPM7, RUNX1T1, ACACA, LMNA, SMAD2, RACGAP1, FLNC, CDKN1B, SP1, PNRC1, FBXL5, SYTL2, C14ORF129, APAF1, IKBKB, SETD2, CLOCK, CLCN4 |
| alternative splicing | 102 | 0.002 | MPZL1, GNPDA2, IL16, GLYATL1, HTRA2, GATA3, BPNT1, SCAMP1, RSBN1L, GTPBP8, STRN3, BCL2L11, BACE1, FBXL5, PCMTD1, TESK2, VGLL4, CHORDC1, EIF2AK4, SNX7, AKAP12, PEX5L, MED12L, MIA3, NIPBL, SFRS16, KIAA1012, SSX2IP, GAD1, IQSEC2, GLT1D1, ESRRA, MAP2K1, CDC23, SMAD2, VAV2, SFMBT1, CDKN1C, CRBN, SEMA6D, LASP1, PARP8, SYTL2, PPARD, ELF2, ATP10B, NDST1, ARHGAP19, KIAA1370, PDIA6, SKAP1, FAM63B, TMEM108, PCBP4, ZFYVE16, RSPO3, DPP6, USP16, AKT3, COX15, AP3B1, SLC22A9, RUNX1T1, TLE4, MBNL2, CBR4, FOXJ3, MBD2, FLNC, GRM1, ZNF335, SEMA4G, C1QL3, KSR1, C1ORF91, MKNK1, KIT, CHD7, ACSL1, CCL20, DGKG, PCMT1, WAC, PCSK6, KIF21A, PPAP2A, FYCO1, ZNF562, BAHD1, RBM24, UPF2, COL4A1, DLGAP2, LMNA, ITPR1, C12ORF41, RGS14, POFUT2, APAF1, SETD2, DUSP8, RNF111 |
| splice variant | 102 | 0.002 | MPZL1, GNPDA2, IL16, GLYATL1, HTRA2, GATA3, BPNT1, SCAMP1, RSBN1L, GTPBP8, STRN3, BCL2L11, BACE1, FBXL5, PCMTD1, TESK2, VGLL4, CHORDC1, EIF2AK4, SNX7, AKAP12, PEX5L, MED12L, MIA3, NIPBL, SFRS16, KIAA1012, SSX2IP, GAD1, IQSEC2, GLT1D1, ESRRA, ACACA, CDC23, SMAD2, VAV2, SFMBT1, CDKN1C, CRBN, SEMA6D, LASP1, PARP8, SYTL2, PPARD, ELF2, ATP10B, NDST1, ARHGAP19, KIAA1370, PDIA6, SKAP1, FAM63B, TMEM108, PCBP4, ZFYVE16, RSPO3, DPP6, USP16, AKT3, COX15, AP3B1, SLC22A9, RUNX1T1, TLE4, MBNL2, CBR4, FOXJ3, MBD2, FLNC, GRM1, ZNF335, SEMA4G, C1QL3, KSR1, C1ORF91, MKNK1, KIT, CHD7, ACSL1, CCL20, DGKG, PCMT1, WAC, PCSK6, KIF21A, PPAP2A, FYCO1, ZNF562, BAHD1, RBM24, UPF2, COL4A1, DLGAP2, LMNA, ITPR1, C12ORF41, RGS14, POFUT2, APAF1, SETD2, DUSP8, RNF111 |
| regulation of cell proliferation | 20 | 0.002 | PPARD, ESRRA, ADAM10, CCL2, TBX2, RXRA, CHEK1, SKI, SMAD2, KIT, MBD2, CDH5, CDKN1C, CUL2, MSX1, CDKN1B, NCK1, BAI1, PPAP2A, FGFBP1 |
| transferase | 27 | 0.004 | FUT9, NDST1, MKNK1, CHEK1, KIT, EPHB3, GLYATL1, DGKG, PCMT1, AKT3, SHMT2, GLT1D1, MAP2K1, TRPM7, PFKP, EPHA4, PANK3, EPHA8, PARP8, PCMTD1, TESK2, POFUT2, SETD2, IKBKB, UTP14C, EIF2AK4, CLOCK |
| atp-binding | 26 | 0.004 | ADCY1, ATP10B, CTPS, MKNK1, CHEK1, KIT, EPHB3, ACSL1, CHD7, DGKG, KIF21A, AKT3, MAP2K1, TRPM7, MSH2, KIF5A, ACACA, PFKP, EPHA4, PANK3, EPHA8, TESK2, APAF1, IKBKB, CLCN4, EIF2AK4 |
| acetylation | 42 | 0.008 | CRTC2, TXN2, CTPS, ARHGAP19, CHCHD3, CUL2, ACSL1, PCMT1, WAC, SLC4A1, C11ORF58, FYCO1, BPNT1, STAG2, AP3B1, RSBN1L, SHMT2, KHDRBS2, PDCD10, MAP2K1, ACTN4, MESDC1, KIF5A, MSH2, ACACA, PFKP, LMNA, CDC23, TLE4, ACTN1, SMAD2, YWHAG, SP1, LASP1, NCK1, SLAIN2, RAB22A, CHORDC1, IKBKB, RAB10, KPNA3, EIF2AK4 |
| nucleotide-binding | 29 | 0.013 | ADCY1, ATP10B, CTPS, MKNK1, CHEK1, KIT, EPHB3, ACSL1, CHD7, DGKG, KIF21A, AKT3, MAP2K1, TRPM7, MSH2, KIF5A, GTPBP8, PFKP, ACACA, EPHA4, PANK3, EPHA8, RAB22A, TESK2, APAF1, RAB10, IKBKB, CLCN4, EIF2AK4 |
| intracellular signaling cascade | 25 | 0.014 | ADCY1, CCL2, NDST1, MKNK1, CHEK1, KIT, STAC, PCBP4, DGKG, DUSP16, PPAP2A, MAP2K1, MSH2, RXRA, CYP26A1, SMAD2, VAV2, RACGAP1, GRM1, RAB22A, RAB10, KSR1, IKBKB, ASB4, DUSP8 |
| disease mutation | 27 | 0.020 | RBM6, KIT, NIPBL, CHD7, HTRA2, BCHE, GATA3, SOX18, PEX13, SLC4A1, KIF21A, EIF2B2, GAD1, AP3B1, COX15, PTPRJ, COL4A1, MAP2K1, ACTN4, MSH2, KIF5A, LMNA, SMAD2, CDKN1C, CYP7B1, LHFPL5, MSX1 |
| ATP binding | 28 | 0.021 | ADCY1, ATP10B, CTPS, MKNK1, CHEK1, KIT, EPHB3, ACSL1, CHD7, DGKG, KIF21A, EIF2B2, AKT3, MAP2K1, TRPM7, MSH2, KIF5A, ACACA, PFKP, EPHA4, PANK3, EPHA8, TESK2, APAF1, KSR1, IKBKB, CLCN4, EIF2AK4 |
| adenyl ribonucleotide binding | 28 | 0.025 | ADCY1, ATP10B, CTPS, MKNK1, CHEK1, KIT, EPHB3, ACSL1, CHD7, DGKG, KIF21A, EIF2B2, AKT3, MAP2K1, TRPM7, MSH2, KIF5A, ACACA, PFKP, EPHA4, PANK3, EPHA8, TESK2, APAF1, KSR1, IKBKB, CLCN4, EIF2AK4 |
| cytoplasm | 48 | 0.025 | CRTC2, GNPDA2, IL16, AKAP12, MKNK1, CHEK1, PEX5L, SKAP1, SPRY1, STAC, PCBP4, DGKG, ZFYVE16, DUSP16, PCMT1, AKT3, IQSEC2, UPF2, RBM24, ACTN4, KIF5A, LDHAL6A, STRN3, ACACA, ACTN1, SMAD2, MBNL2, RACGAP1, FLNC, YWHAG, PANK3, CRBN, CDKN1B, SP1, SEMA6D, LASP1, NCK1, FBXL5, PCMTD1, SYTL2, C14ORF129, APAF1, KSR1, IKBKB, KPNA3, DUSP8, CLOCK, RNF111 |
| organelle membrane | 20 | 0.032 | SCAMP1, SHMT2, SLC9A6, LMNA, CHCHD3, CYP26A1, HERC2, ATP5G3, ITPR1, BCL2L11, CYP7B1, MIA3, ACSL1, HTRA2, NCK1, ZFYVE16, PEX13, GAD1, PMPCB, COX15 |
| nucleoside binding | 29 | 0.034 | ADCY1, ATP10B, CTPS, MKNK1, CHEK1, KIT, EPHB3, ACSL1, CHD7, DGKG, KIF21A, EIF2B2, AKT3, ACTN4, MAP2K1, TRPM7, MSH2, KIF5A, PFKP, ACACA, EPHA4, PANK3, EPHA8, TESK2, APAF1, KSR1, IKBKB, CLCN4, EIF2AK4 |
| nucleus | 58 | 0.039 | PPARD, ELF2, IL16, ARHGAP19, RBM6, SKAP1, GATA3, SOX18, USP16, STAG2, RSBN1L, KHDRBS2, ACTN4, RXRA, RUNX1T1, TLE4, MBNL2, FOXJ3, MBD2, ZNF335, MSX1, NCK1, TESK2, VGLL4, KPNA3, CLOCK, CRTC2, IRX5, MKNK1, CHEK1, MED12L, CHD7, NIPBL, SFRS16, DUSP16, WAC, SSX2IP, ZNF562, BAHD1, ESRRA, RBM24, CEBPB, TBX2, MSH2, LMNA, SKI, SMAD2, RACGAP1, CDKN1C, CDKN1B, SP1, PNRC1, HIVEP2, SETD2, DUSP8, ZNF572, UTP14C, RNF111 |
| adenyl nucleotide binding | 28 | 0.044 | ADCY1, ATP10B, CTPS, MKNK1, CHEK1, KIT, EPHB3, ACSL1, CHD7, DGKG, KIF21A, EIF2B2, AKT3, MAP2K1, TRPM7, MSH2, KIF5A, ACACA, PFKP, EPHA4, PANK3, EPHA8, TESK2, APAF1, KSR1, IKBKB, CLCN4, EIF2AK4 |
| membrane-enclosed lumen | 29 | 0.049 | TXN2, PDIA6, CHEK1, KIT, MED12L, HTRA2, BCHE, ENTPD5, WAC, PCSK6, SHMT2, MRPL3, CEBPB, ACTN4, TBX2, STRN3, LMNA, CDC23, ACTN1, SMAD2, SKI, CBR4, MBD2, ITPR1, CDKN1C, SP1, UTP14C, PMPCB, CLOCK |
| **Profile 18** | | | |
| metal-binding | 36 | 4.552E-4 | ZMYND11, ADCY7, ZNF367, ZEB1, NR2C1, ACVR1C, PGR, PTER, PCGF5, GPHN, MAP3K2, ADH4, ZNF396, FGD6, DDAH1, ZNF423, RBM26, AEBP2, CARS, ZNF644, NUDT12, LPP, ZFP30, SNAI1, PCLO, ZDBF2, MARCH1, RNF180, PGM3, CYBB, TAF15, MEP1A, NLN, RNF26, ACVR1, TRIT1 |
| phosphoprotein | 67 | 0.001 | ENAH, GABRB2, UVRAG, IQGAP2, ZEB1, UBQLN1, RAB1A, GLDC, PGR, SRRM2, SNTB1, QKI, STK39, PTPRK, CARS, TTC33, LYN, ZNF644, MED13, RFTN2, PCLO, ZDBF2, RAD51, PGM3, BRWD1, TAF15, IPO7, GTF2I, C14ORF101, USP25, DCUN1D5, ACVR1, ZMYND11, MFSD6, SLC38A2, SSFA2, USP4, AHCTF1, ZNF367, NR2C1, PHIP, IGSF11, GPHN, MAP3K2, ARMCX3, SH3TC2, FGD6, C1ORF96, ZNF423, RBM26, AEBP2, PTPN9, KIF3A, LPP, BECN1, YTHDC2, SNAI1, RSBN1, NMT2, TEX15, PPID, NLN, USP48, PLA2G4C, PAPOLG, TRIT1, SSR3 |
| zinc | 27 | 0.002 | ZMYND11, ZNF367, ZEB1, NR2C1, PGR, PCGF5, ADH4, ZNF396, FGD6, DDAH1, RBM26, ZNF423, AEBP2, CARS, ZNF644, LPP, ZFP30, PCLO, SNAI1, ZDBF2, MARCH1, RNF180, TAF15, MEP1A, NLN, RNF26, TRIT1 |
| cytoplasm | 36 | 0.003 | PPP4R4, BCAT1, ENAH, SSFA2, USP4, UVRAG, AHCTF1, UBQLN1, KLHL2, PGR, GPHN, KLHL5, MAP3K2, BTBD1, ADH4, SNTB1, ZNF396, QKI, STK39, FGD6, ARHGEF3, CARS, PTPN9, LYN, BECN1, LPP, DDIT4, NMT2, BRWD1, IPO7, GTF2I, PPID, NLN, SMURF2, USP48, TRIT1 |
| zinc-finger | 21 | 0.010 | AEBP2, ZMYND11, ZNF644, ZFP30, ZNF367, ZEB1, SNAI1, PCLO, ZDBF2, NR2C1, MARCH1, PGR, RNF180, PCGF5, TAF15, ZNF396, RNF26, FGD6, ZNF423, TRIT1, RBM26 |
| zinc ion binding | 28 | 0.012 | ZMYND11, ZNF367, ZEB1, NR2C1, PTER, PGR, PCGF5, ADH4, ZNF396, FGD6, DDAH1, ZNF423, RBM26, AEBP2, CARS, ZNF644, LPP, ZFP30, PCLO, SNAI1, ZDBF2, MARCH1, RNF180, TAF15, MEP1A, NLN, RNF26, TRIT1 |
| transition metal ion binding | 32 | 0.013 | ZMYND11, ZNF367, ZEB1, NR2C1, ACVR1C, PGR, PTER, PCGF5, ADH4, ZNF396, FGD6, DDAH1, ZNF423, RBM26, AEBP2, CARS, NUDT12, ZNF644, LPP, ZFP30, PCLO, SNAI1, ZDBF2, MARCH1, RNF180, CYBB, TAF15, MEP1A, NLN, RNF26, ACVR1, TRIT1 |
| nucleotide-binding | 20 | 0.017 | CARS, KIF3A, GCLC, LYN, RAB5B, ADCY7, YTHDC2, RAB1A, ACVR1C, RAD51, GPHN, DIRAS3, MAP3K2, RAB22A, ABCB10, STK39, PAPOLG, ACSL6, TRIT1, ACVR1 |
| ion binding | 43 | 0.026 | ZMYND11, GCLC, SLC38A2, ADCY7, GABRB2, ZNF367, ZEB1, NR2C1, ACVR1C, PGR, PTER, PCGF5, GPHN, MAP3K2, ADH4, SNTB1, ZNF396, FGD6, DDAH1, ACSL6, ZNF423, RBM26, AEBP2, CARS, ZNF644, NUDT12, LPP, ZFP30, SLC10A2, SNAI1, PCLO, ZDBF2, MARCH1, RNF180, PGM3, CYBB, TAF15, DLL4, MEP1A, NLN, RNF26, ACVR1, TRIT1 |
| metal ion binding | 42 | 0.028 | ZMYND11, GCLC, SLC38A2, ADCY7, ZNF367, ZEB1, NR2C1, ACVR1C, PGR, PTER, PCGF5, GPHN, MAP3K2, ADH4, SNTB1, ZNF396, FGD6, DDAH1, ACSL6, ZNF423, RBM26, AEBP2, CARS, ZNF644, NUDT12, LPP, ZFP30, SLC10A2, SNAI1, PCLO, ZDBF2, MARCH1, RNF180, PGM3, CYBB, TAF15, DLL4, MEP1A, NLN, RNF26, ACVR1, TRIT1 |
| cation binding | 42 | 0.032 | ZMYND11, GCLC, SLC38A2, ADCY7, ZNF367, ZEB1, NR2C1, ACVR1C, PGR, PTER, PCGF5, GPHN, MAP3K2, ADH4, SNTB1, ZNF396, FGD6, DDAH1, ACSL6, ZNF423, RBM26, AEBP2, CARS, ZNF644, NUDT12, LPP, ZFP30, SLC10A2, SNAI1, PCLO, ZDBF2, MARCH1, RNF180, PGM3, CYBB, TAF15, DLL4, MEP1A, NLN, RNF26, ACVR1, TRIT1 |
